# Virtual Screening of Acetylcholinesterase Inhibitors Using the Lipinski's Rule of Five and ZINC Databank

**DOI:** 10.1155/2015/870389

**Published:** 2015-01-22

**Authors:** Pablo Andrei Nogara, Rogério de Aquino Saraiva, Diones Caeran Bueno, Lílian Juliana Lissner, Cristiane Lenz Dalla Corte, Marcos M. Braga, Denis Broock Rosemberg, João Batista Teixeira Rocha

**Affiliations:** ^1^Departamento de Bioquímica e Biologia Molecular, Programa de Pós-Graduação em Ciências Biológicas: Bioquímica Toxicológica, Centro de Ciências Naturais e Exatas, Universidade Federal de Santa Maria, 97105-900 Santa Maria, RS, Brazil; ^2^Grupo de Estudos em Bioquímica, Farmacologia e Toxicologia Molecular, Unidade Acadêmica de Serra Talhada, Universidade Federal Rural de Pernambuco, 56900-000 Serra Talhada, PE, Brazil

## Abstract

Alzheimer's disease (AD) is a progressive and neurodegenerative pathology that can affect people over 65 years of age. It causes several complications, such as behavioral changes, language deficits, depression, and memory impairments. One of the methods used to treat AD is the increase of acetylcholine (ACh) in the brain by using acetylcholinesterase inhibitors (AChEIs). In this study, we used the ZINC databank and the Lipinski's rule of five to perform a virtual screening and a molecular docking (using Auto Dock Vina 1.1.1) aiming to select possible compounds that have quaternary ammonium atom able to inhibit acetylcholinesterase (AChE) activity. The molecules were obtained by screening and further *in vitro* assays were performed to analyze the most potent inhibitors through the IC_50_ value and also to describe the interaction models between inhibitors and enzyme by molecular docking. The results showed that compound D inhibited AChE activity from different vertebrate sources and butyrylcholinesterase (BChE) from *Equus ferus* (*Ef*BChE), with IC_50_ ranging from 1.69 ± 0.46 to 5.64 ± 2.47 *µ*M. Compound D interacted with the peripheral anionic subsite in both enzymes, blocking substrate entrance to the active site. In contrast, compound C had higher specificity as inhibitor of *Ef*BChE. In conclusion, the screening was effective in finding inhibitors of AChE and BuChE from different organisms.

## 1. Introduction

Alzheimer's disease (AD) was first reported by the pathologist Alois Alzheimer in 1907. It is a neurological disorder characterized by a significant decrease in hippocampal and cortical levels of the neurotransmitter acetylcholine (ACh) [1] with formation of extracellular amyloid plaques and intracellular neurofibrillary tangles that lead to neurotoxicity [2]. The AD affects up to 5% of people over 65 years, rising to 20% of those over 80 years [3]. One of the major therapeutic strategies adopted for AD treatment is based on the cholinergic hypothesis. Clinically, AD is associated with cognitive, functional, and behavioral symptoms, which can be explained by the cholinergic neurotransmission deficit with the loss of cholinergic neurons [4].

The neurotransmitter ACh plays a key role in learning and memory processes by activating nicotinic and muscarinic receptors of the central nervous system (CNS) [5]. The acetylcholinesterase (AChE) is an enzyme that hydrolyzes ACh to acetate and choline in the synaptic cleft, terminating the ACh neurotransmission [6]. The inhibitory effect on AChE activity increases ACh in synaptic cleft with overactivation of the cholinergic transmission [7]. Since disruption of cholinergic neurotransmission is involved in different brain functions, the search for new acetylcholinesterase inhibitors (AChEIs) is relevant as an early step to select molecules that can be used in preclinical trials as potential pharmacological agents or even to synthesize other kinds of compounds, such as insecticides.

Virtual screening is established as an effective method for filtering compounds in the course of new drug discovery [8]. During this long process, it is possible to search for compounds with specific features that can, potentially, lead to the development of effective therapeutic agents. For instance, the consideration of the Lipinski's rule of five [9], that is, molecular weight lower than 500 Da, number of donor hydrogen bonds less than 5, number of acceptor hydrogen bonds less than 10, and the *x*log⁡⁡*P* lower than 5, is of importance for the screening of drugs with pharmacological activity.

The molecular docking is a method that can predict the most favorable orientation of a molecule (ligand) when interacting with a macromolecular target, such as an enzyme or a receptor, to form a stable complex. The crucial thermodynamic parameter involved in this method is the binding free energy (Δ*G*_binding_), which checks the theoretical stability of the ligand-protein complex [10]. Based on this principle, the main objective of the present study was to propose a strategy of virtual screening to unravel potential new AChEIs by using the virtual molecules ZINC bank and selecting compounds that obey the Lipinski's rule of five which have quaternary ammonium atom (because of the similarity with ACh). We also assessed the inhibitory activity of selected compounds* in vitro*, in order to check which compounds have the highest inhibitory activity using different AChE sources, purified AChE from* Electrophorus electricus* (*Ee*AChE), AChE from* Danio rerio *(*Dr*AChE), and human AChE (*Hs*AChE). Furthermore in order to verify whether the selected compounds could also inhibit butyrylcholinesterase (BChE) activity, we investigated the effects of these compounds on purified BChE from* Equus ferus *(*Ef*BChE).

## 2. Materials and Methods

### 2.1. *In Silico* Analysis

To search for new drugs with binding affinity to AChE, we used the virtual molecules ZINC bank (http://zinc.docking.org/), where approximately 5.5 million of different molecular structures are deposited [11]. First, we selected only tridimensional structures of compounds with quaternary ammonium atom that were in accordance with the Lipinski's rule of five. In addition, another rule was also included: the number of rotatable bonds had to be less than 10 [12]. The molecules obtained were downloaded and their geometry optimized using the software Avogadro 0.9.4 following the MMFF94 method.

The molecular docking simulation was used as a second screening, aiming to search for compounds with higher inhibitory capacity and to propose an interaction model. We used different crystallographic structures of AChE from Protein Data Bank (PDB) (http://www.pdb.org/). The CHIMERA 1.5.3 software was used to remove molecules, ions, and water and to minimize the structure of proteins, using the Gasteiger charges with 500 steps of minimization.

After obtaining the ligands and enzymes, their structures were converted to pdbqt format, using the Auto Dock Tools 1.5.4 program, in which all the rotatable bonds of ligands were allowed to rotate freely, and the receptors were considered rigid. For docking studies, we used the Auto Dock Vina 1.1.1 [13], with 1 Å of spacing between the grid points. The grid box was centered on the active site of the enzymes with high resolution, allowing the program to search for additional places of probable interactions between the ligands and the receptor. Other configurations were considered default.

**Figure 1:**
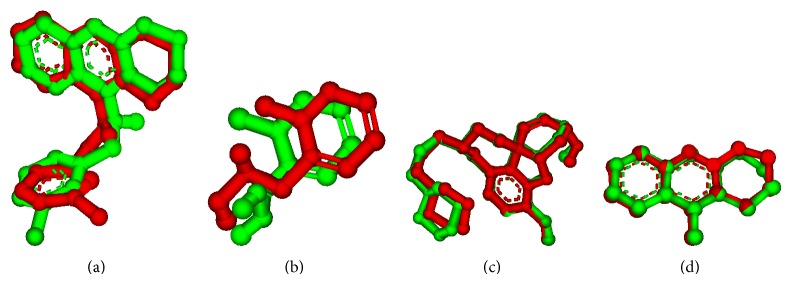
Molecular overlapping of the crystal ligands (red) and the best pose of ligands proposed by Auto Dock Vina 1.1.1 program (green), for the enzymes 1QON (a), 2VQ6 (b), 3I6 M (c), and 2BDS (d). The nonpolar hydrogen atoms were omitted.

The type of enzyme, species, PDB code, RMSD value, coordinates, and size of the grid box are shown in Table 1. Importantly, some enzymes do not present the RMSD value because they do not have inhibitor on their structures. The figures of structures with RMSD are represented in Figure 1. The RMSD value (less than 2 Å) is a criterion often used for correcting bound structure prediction [14]. The redockings were performed with the same configurations of the previous performed dockings.

For* in vitro *assays, we selected the compounds that presented lower binding energy (Δ*G*_binding_) in all enzymes used for the screening. The interactions between ligand-protein were visualized by Accelrys Discovery Studio Visualizer 2.5.

### 2.2. *In Vitro* Analysis

The compounds selected as inhibitors of AChE activity were obtained commercially from MolPort (http://www.molport.com/buy-chemicals/index). They were dissolved in dimethyl sulfoxide (DMSO), at a final concentration of 0.1%.

The cholinesterase activities were measured based on Ellman et al.'s method [15]. The increase of absorbance was monitored at 412 nm in a reaction mixture containing 10 mM potassium phosphate buffer, pH 7.4, and 1 mM DTNB [5,5′-dithiobis-(2-nitrobenzoic) acid] (from Sigma) in the presence of one of the following enzymes: purified AChE from* Electrophorus electricus* (*Ee*AChE)—0.05 U/ml, AChE from human erythrocytes (*Hs*AChE), AChE from* Danio rerio* (*Dr*AChE)—0.5 *μ*g, and purified BChE from* Equus ferus* (*Ef*BChE)—0.05 U/mL. The compounds (or only 0.1% DMSO for the control group) were preincubated with the enzyme during 10 minutes at room temperature, and the reaction was started with the addition of acetylthiocholine or butyrylthiocholine (0.8 mM).

Haemoglobin-free erythrocyte ghosts were prepared according to the method previously described [16]. Blood of nonfasted healthy voluntary donors was collected. Heparinized human blood was centrifuged at 3000 g for 10 min. The packed erythrocytes were diluted in 20 volumes (w/v) of hypotonic sodium/potassium phosphate buffer (6.7 mM, pH 7.4) to facilitate the hemolysis, followed by centrifugation at 30.000 g for 30 min at 4°C. The supernatant was removed and the pellet resuspended in hypotonic phosphate buffer. After two additional washing cycles, the pellet was resuspended in sodium/potassium phosphate buffer (0.1 M, pH 7.4) and then centrifuged again at 30.000 g for 30 min at 4°C. The supernatant was gently removed and the pellet was stored. Aliquots of the erythrocyte ghosts were stored at −20°C until usage within one week. The sample was diluted 10 times for AChE activity measurement. Fifty *μ*L of the stored ghost preparation, in a final volume of 200 *μ*L, was used for the assay. Hemoglobin content from ghost membranes was measured at 540 nm as the cyano-met-Hb form, but no hemoglobin was detected.

The* Dr*AChE assay was performed as previously described by Rosemberg et al. (2010) [17]. Briefly, zebrafish brains were homogenized on ice in 60 volumes (v/w) of Tris-citrate buffer (50 mM Tris, 2 mM EDTA, 2 mM EGTA, and pH 7.4, with citric acid) using a Potter-Elvehjem-type glass homogenizer. Samples (0.5 *μ*g protein) were preincubated for 10 min at 25°C and the enzyme activity was further assessed in the absence and the presence of the selected compounds.

### 2.3. Statistical Analysis

The IC_50_ values were determined by nonlinear regression (log concentration-inhibition curves). Data were analyzed by one-way analysis of variance (ANOVA) followed by Student-Newman-Keuls test. Statistical significance was set at *P* < 0.05. The statistics have been performed using GraphPad Prism 5 (version 5.01, GraphPad Software, Inc., USA).

## 3. Results and Discussion

**Figure 2:**
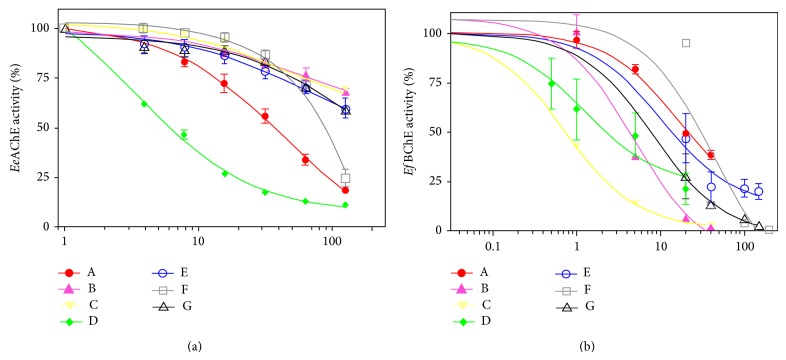
Inhibition of purified AChE activity from* Electrophorus electricus *(a) and BChE activity from* Equus ferus* (b), by the seven compounds selected by virtual screening.

The first virtual screening retrieved 382 compounds that obey the Lipinski's rule of five and have the ammonium quaternary atom. The retrieved compounds were docked with the enzymes listed in Table 1 (second screening). We obtained the mean value of the lower binding free energy for each molecule resulting in 7 compounds (Table 2). These compounds were further obtained commercially for* in vitro* assay. The* in vitro *assay was carried out as a third screening step, in which we identified that the compound “D” presented the higher anti-AChE activity (for* Ee*AChE,* Dr*AChE, and* Hs*AChE), being also able to inhibit the* Ef*BChE. These results suggest that the respective compound is not specific to acetylcholinesterases, because its IC_50_ was very similar to all the enzymes tested in this study, with values ranging from 1.69 ± 0.46 to 5.64 ± 2.47. On the other hand, the compound “C” presented a higher inhibitory potency against* Ef*BChE, with an IC_50_ value of 0.75 ± 0.18, than with AChEs IC_50_ values ranging from 92.08 ± 39.73 to 761.17 ± 127.6. These data demonstrate that even if a strategy is adopted to select specific AChEIs using* in silico* analysis, it is relevant to assess whether the potential inhibitors may also alter BChE activity* in vitro*. The IC_50_ values for both enzymes tested are shown in Table 3 and the graphics for purified* Ee*AChE and* Ef*BChE are depicted in Figure 2.

**Figure 3:**
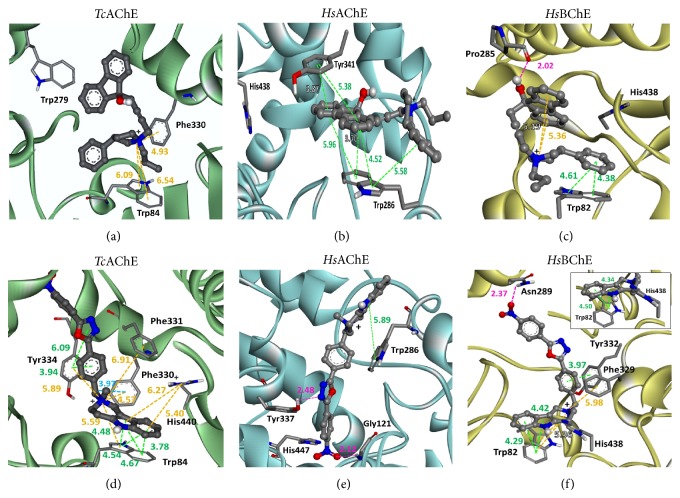
The interactions between C and D compounds with* Tc*AChE (1EA5, enzyme in green),* Hs*AChE (1B41, enzyme in blue), and* Hs*BChE (4BDS, enzyme in yellow) obtained after molecular docking. The compound “C” is shown in (a), (b), and (c), and the “D” in (d), (e), and (f), respectively. The nonpolar hydrogen atoms were omitted; the nitrogen atoms are represented in blue, oxygen in red, carbon in gray, and the polar hydrogen in white. The inhibitors are represented as ball and stick and the amino acids residues as sticks. The types of interactions are represented by dotted lines with their respective distance, differentiated by colors: *π*-*π* interactions (green), cation-*π* (orange), *σ*-*π* (light blue), and hydrogen bonds (pink).

We further proposed an interaction model for the compounds with AChEs enzymes (*Tc*AChE: PDB: 1EA5 and* Hs*AChE: PDB: 1B41) and* Hs*BChE (PDB: 2BDS), to compare the interactions of the compounds in each enzyme and to investigate the putative mechanisms of inhibition (Figure 3). The* Tc*AChE and compound “C” (Figure 3(a)) have only two cation-*π* interactions (in orange) with Trp84, indicating a lower affinity with the enzyme, and this fact was confirmed by its IC_50_ value for* Ee*AChE (761.17 ± 127.6 *μ*M). However, for human AChE (Figure 3(b)), we found a larger number of *π*-*π* interactions (in green) of Tyr341 and Trp286 (peripheral anionic subsite) with the molecule “C,” indicating a slight increase in affinity and a decrease in the IC_50_ value (92.08 ± 39.73 *μ*M). For* Hs*BChE (Figure 3(c)) it was possible to detect one hydrogen bond (H-bond) between the compound “C” and Pro285. This H-bond is stronger when compared with the *π*-*π* or cation-*π* interactions. Furthermore, the results showed that *π*-*π* and cation-*π* interactions occur stacking between the compound “C” and the enzyme in the anionic subsite (Trp82). All these interactions could explain, at least partially, the high inhibitory potency of the molecule “C” for* Hs*BChE (IC_50_ = 0.75 ± 0.18 *μ*M). These results indicated that the compound “C” is more specific to* Hs*BChE.

On the other hand, the molecule “D” was able to inhibit the* Ee*AChE,* Dr*AChE*, Hs*AChE, and* Hs*BChE with a similar potency (see Table 3). The docking results suggest that for* Tc*AChE occur a larger number of *π*-*π* and cation-*π* interactions, mainly with the anionic subsite (Trp84), catalytic triad (His440), and peripheral anionic subsite (Tyr334)—Figure 3(d). In the presence of the compound “D,” a very similar conformation was detected for* Hs*BChE (Figure 3(f)) but with one H-bond between Asn289 and nitro moiety from “D.” This same nitro moiety has an important role in* Hs*AChE, making one H-bond with Gly121 (oxyanion hole). Another H-bond occurs between oxadioazole group and Tyr337 (peripheral anionic subsite) (Figure 3(e)). In addition, a *π*-*π* stacking was observed between Trp286 (peripheral anionic subsite) and the compound “D.” The different model of which “D” interacts with the* Hs*AChE (Figure 3(e)) could be responsible for its more potent inhibitory effect on the enzyme.

**Figure 4:**
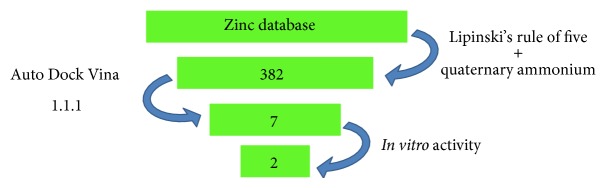
Scheme of the virtual screening used in this study. The strategy excludes a large number of compounds for further* in vitro* assays, as well as the costs and working time.

In all these models proposed above, the interactions of inhibitors with ChEs are expected to prevent the entrance of ACh in the activity site from AChEs and* Hs*BChE, consequently, causing their inhibition. It is possible to observe that both compounds (C and D) interact rather with peripheral anionic and anionic subsites, probably due to the presence of aromatic residues in both peripheral anionic and anionic subsites. Importantly, similar observations were made by other studies involving AChEIs and molecular modeling [18, 19]. The IC_50_ values found for the compounds “C” and “D” are in the range of *μ*M; that is, they are 1–3 orders of magnitude higher than those of tacrine and donepezil (IC_50_ = 205 ± 18 nM and 11.6 ± 1.6 nM, resp. [19]), which are two commercial drugs for treating AD. Moreover, according the thermodynamic data (Δ*G*_binding_), we observed that no type of correlation occurred with the IC_50_ values. An overall scheme of the strategy used for this study is depicted in Figure 4.

## 4. Conclusion

In this study, we carried out a total of tree hierarchical screening steps (two* in silico* and one* in vitro*) in order to search for potential molecules able to act as AChEIs. We found one compound “D” with relevant anti-AChE and anti-BChE activity. To our surprise, we found one molecule “C” which inhibited* Ef*BChE more significantly than it did with AChEs. These results suggest that selecting compounds with pharmacophoric properties (Lipinski's rule of five) and performing the molecular docking screening to search potential inhibitors are interesting strategies that could be used for high throughput screenings aiming to detect new compounds with desirable biological activity. We also reported the importance of aromatics rings in the inhibitors. These aromatic moieties in the ligands perform *π*-*π* and cation-*π* interactions with several aromatic residues located in the gorge of AChE (for instance, Trp84, Trp279, Phe330, Phe331, and Tyr334). These molecules interact at the peripheral anionic subsite and anionic subsite of AChE, preventing the hydrolysis of ACh. Despite the fact that some compounds act as inhibitors of AChE and BChE, we emphasize that other approaches, such as* in vivo* studies, are necessary to validate the pharmacological and toxicological properties of these compounds.

## Figures and Tables

**Table 1:** Information about the AChE enzymes: species, PDB code, coordinates and size of grid box, and RMSD value.

Species	PDB code	Coordinates of grid box	Size of grid box	RMSD value (Å)
*Drosophila melanogaster *	1DX4	*x*: 30.311	*x*: 44	
		*y*: 70.424	*y*: 48	—
		*z*: 10.617	*z*: 52	
	1QO9	*x*: 28.259	*x*: 44	
		*y*: 70.453	*y*: 52	—
		*z*: 12.592	*z*: 44	
	1QON	*x*: 29.403	*x*: 40	1.03
		*y*: 71.508	*y*: 44	
		*z*: 11.697	*z*: 40	

*Torpedo californica *	2VQ6	*x*: 5.254	*x*: 48	
		*y*: 65.479	*y*: 44	1.94
		*z*: 64.028	*z*: 48	
	3I6M	*x*: 2.092	*x*: 56	
		*y*: 64.337	*y*: 44	0.41
		*z*: 64.75	*z*: 48	
	1EA5	*x*: 4.957	*x*: 48	
		*y*: 64.084	*y*: 44	—
		*z*: 65.094	*z*: 48	

*Mus musculus *	1J06	*x*: 32.473	*x*: 48	
		*y*: 20.287	*y*: 40	—
		*z*: 10.477	*z*: 32	

*Homo sapiens *	3LII	*x*: 91.443	*x*: 52	
		*y*: 88.69	*y*: 40	—
		*z*: −5.859	*z*: 48	
	1B41	*x*: 120.139	*x*: 32	—
		*y*: 108.623	*y*: 52	
		*z*: −132.452	*z*: 36	

*BChE *	2BDS^*^	*x*: 133.076	*x*: 42	0.38
*Homo sapiens *		*y*: 116.113	*y*: 48	
		*z*: 41.093	*z*: 48	

^*^This structure was not used in the virtual screening.

**Table 2:** The seven compounds obtained by molecular docking screening and their features.

Compound (ZINC cod)	Structure	Molecular weight^*^ (g·mol^−1^)	H-bond donor^*^	H-bond acceptor^*^	*x*log⁡P^*^	Rotatable bonds^*^
A (1249551)	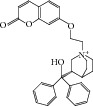	482.6	1	5	1.1	7
B (1280061)	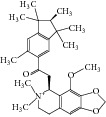	478.6	0	5	2.42	4
C (1771471)	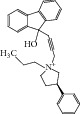	422.6	1	2	2.24	5
D (2417539)	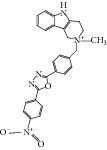	466.5	1	8	1.73	5
E (4311794)	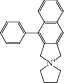	300.4	0	1	1.03	1
F (4372347)	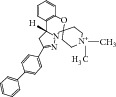	424.6	0	4	1.73	2
G (4937122)	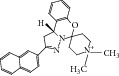	398.5	0	4	1.12	1

^*^Data from ZINC databank.

**Table 3:** The IC_50_ values and Δ*G*_binding_ to the compounds A–G.

Compound	IC_50_ (*µ*M)				Δ*G* (kcal·mol^−1^)		
	*Ee*AChE	*Dr*AChE	*Hs*AChE	*Ec*BChE	*Tc*AChE (1EA5)	*Hs*AChE (1B41)	*Hs*BChE (4BDS)
A	36.33 ± 3.14	26.23 ± 3.98	13.22 ± 1.38	22.22 ± 1.06	−11.7	−9.0	−10.6
B	577.7 ± 84.3	21.16 ± 1.44	37.55 ± 1.97	4.02 ± 0.78	−12.2	−9.8	−11.6
C	761.17 ± 127.6	239.45 ± 2.95	92.08 ± 39.73	0.75 ± 0.18	−11.8	−9.9	−10.6
D	5.39 ± 0.55	2.31 ± 0.29	1.69 ± 0.46	5.64 ± 2.47	−13.0	−10.9	−11.4
E	341.6 ± 54.6	26.41 ± 5.30	512.25 ± 300.65	12.66 ± 7.66	−10.8	−10.2	−9.7
F	84.16 ± 6.0	191.7 ± 29.8	150.95 ± 61.68	32.85 ± 3.88	−11.6	−11.2	−10.6
G	344.39 ± 50.7	49.78 ± 15.28	70.03 ± 7.96	9.51 ± 6.94	−12.6	−11.3	−10.6

*Ee*AChE = AChE from *Electrophorus electricus*; *Dr*AChE = AChE from *Danio rerio*; *Tc*AChE = AChE from *Torpedo californica*; *Hs*AChE and *Hs*BChE = AChE and BChE from *Homo sapiens*, respectively.
